# Microsurgical Reconstruction of Foot Defects: A Case Series with Long-Term Follow-Up

**DOI:** 10.3390/healthcare10050829

**Published:** 2022-04-30

**Authors:** David Breidung, Panagiotis Fikatas, Patrick Mandal, Maresa D. Berns, Andrè A. Barth, Moritz Billner, Ioannis-Fivos Megas, Bert Reichert

**Affiliations:** 1 Department of Plastic, Reconstructive and Hand Surgery, Center for Severe Burn Injuries, Paracelsus Medical University, Klinikum Nürnberg, Breslauer Str. 201, 90471 Nuremberg, Germany; david.breidung@icloud.com (D.B.); patrick.mandal@tirol-kliniken.at (P.M.); maresa.berns@klinikum-nuernberg.de (M.D.B.); andre.barth@klinikum-nuernberg.de (A.A.B.); moritz.billner@klinikum-nuernberg.de (M.B.); bert.reichert@klinikum-nuernberg.de (B.R.); 2 Department of Surgery, Campus Charité Mitte and Campus Virchow-Klinikum, Charité-Universitätsmedizin, Corporate Member of Freie Universität Berlin, Humboldt-Universität zu Berlin and Berlin Institute of Health, Augustenburger Platz 1, 13353 Berlin, Germany; panagiotis.fikatas@charite.de; 3 Department of Plastic, Reconstructive and Aesthetic Surgery, Innsbruck Medical University, Anichstrasse 35, A-6020 Innsbruck, Austria

**Keywords:** foot, reconstructive surgical procedures, microsurgery, free flap, limb salvage

## Abstract

(1) Background: Microsurgical reconstruction of foot defects with free flaps is rare as it is a challenging task for a surgeon. For extensive defects, advanced surgical procedures, such as free flap transfer with microsurgical anastomosis, may be the last chance to avoid major amputation. The aim of the study was to examine the opportunities and risks posed by free flap reconstruction of foot defects and to illustrate in which situations reconstruction is useful on the basis of case characteristics. (2) Methods: In this study, we retrospectively analyzed data of cases with free flap reconstruction of the foot from 2007 to 2022. Therefore, demographic data, comorbidities, information about the defect situation, data on the operational procedure, and complications were evaluated. (3) Results: A total of 27 cases with free flap coverage of foot defects could be included. In 24 of these cases (89%), defect coverage was successful. In 18 patients, some form of complication occurred in the postoperative stage. The most frequently used flap was the latissimus dorsi flap, with 13 procedures. (4) Conclusions: Foot reconstruction using free flaps is a proven procedure for the treatment of larger foot defects and can offer a predominantly good functional outcome. The lengthy process and possible complications should be thoroughly discussed in advance so as to provide criteria, suitably adjusted to the individual prerequisites of the patients, for deciding whether limb salvage using advanced surgical procedures should be attempted.

## 1. Introduction

The foot itself and problems of the foot are closely related to quality of life [1]. The prevalence of foot problems is high and foot problems, especially in women, show a strong correlation with quality of life and specific parameters, such as foot function and foot pain [2]. Therefore, preserving the foot with all its capabilities and eliminating foot problems is an important factor in improving the overall well-being of affected patients.

Reconstruction of foot defects, particularly in the weight-bearing plantar area, can be extremely challenging for the surgeon. Histologically, the epidermal mean thickness at the sole significantly exceeds the thickness in other anatomical regions (1.4 mm vs. 0.1 mm) [3,4]. Shock absorption and spreading of compressive forces of the plantar foot is achieved by subcutaneous adipose tissue lobules [3,5]. In load distribution analysis it was shown that the heel is responsible for 60% of the weight-bearing load [6]. Meanwhile, the forefoot carries 28% of the weight-bearing load, the midfoot carries 8%, and the toes are only marginally involved. These local peculiarities make the sole of the foot an extremely challenging recipient site. Reconstruction of extensive defects of the dorsal foot also presents as a very demanding area. A thin gliding surface for extensor tendons must be restored without substantially altering the shape of the foot [7]. Finally, the results should not only match functional but also aesthetic measures, as well as ensuring the ability to wear regular footwear after surgery [7,8].

Prior to this study, several studies were conducted on the subject of foot reconstruction with predominantly successful results. In 1994, Harris et al. presented a study in a pediatric population with coverage of weight-bearing defects [9]. Here, all 13 reconstructive surgeries were initially successful; however, two additional free flap coverages were required during the course of treatment due to pressure necrosis. Liebau et al. compared predominantly free flap techniques based on 59 plantar reconstructions performed and proved good functional results in weight-bearing patterns [10]. Functional limitations, such as the need to wear customized shoes, would potentially have to be accepted for limb salvage [11]. In 2018, Crowe et al. presented a systematic review of foot reconstruction that included 98 studies with free tissue transfer and presented an algorithm for foot reconstruction and flap selection [3]. In general, skin grafts using local or regional flaps or free flap reconstruction can be used to cover foot defects. In extensive complicated defects, an advanced surgical procedure such as free flap coverage by an experienced surgeon is a good option to avoid amputation of the lower extremity.

In this study, we investigated the courses of treatment given to patients with such defects treated in our Department of Plastic, Reconstructive and Hand Surgery, Center for Severe Burn Injuries of Klinikum Nürnberg, Nuremberg, Germany. Therefore, we focused on differences in flap-survival, postoperative complications and functional outcomes for these patients compared to those in the literature. The aim of this study was to examine, on the basis of our own results, whether foot reconstruction using a free flap transfer is a suitable procedure for foot defects with respect to associated benefits and risks, and thereby to illustrate in which situations reconstruction is useful with regard to the case characteristics.

## 2. Materials and Methods

The patient-specific data of reconstructive surgeries of the foot with free flap procedures at the Department of Plastic, Reconstructive and Hand Surgery, Center for Severe Burn Injuries of Klinikum Nürnberg, from June 2007 to January 2022 were obtained from our database. The department has approximately 1300 inpatient cases annually and the Nuremberg metropolitan region has about 3,600,000 inhabitants. In addition to the cases operated on in our clinic, we also included a patient who underwent reconstructive surgery in 2000 at the Clinic of Plastic Surgery, University Medical Center Schleswig-Holstein, Campus Lübeck. The patient in question was subsequently followed-up by his surgeon at Klinikum Nürnberg. Cases were analyzed for demographic data, comorbidities, information concerning the defect site, timing of reconstruction, procedure of surgical reconstruction, and postoperative outcome. The study was conducted in accordance with the Declaration of Helsinki. Because of the retrospective design of this study, ethical review and approval were waived.

The indication for reconstruction with free flaps was made according to the hospital’s internal protocol. The inclusion criteria were the reconstructive coverage with a free flap of the foot defect. Patients who had already been operated on with a free flap at a different hospital were not included in the study. Exclusion criteria also comprised any form of direct wound closure, skin grafts, local and regional flap techniques, and reconstruction with dermis substitutes for foot reconstruction. Other previous surgeries, such as debridement or osteosynthesis, did not result in exclusion from the study. No selection was made for age, comorbidities, or other case-characterizing factors. Cases were evaluated independently by two investigators using discharge letters, operative reports, and admission forms. An overview of the cases was obtained by searching the hospital database for the surgical procedures performed.

The data were collected and analyzed using Excel^®^ (Microsoft, Redmond, USA). Categorical variables were reported as total numbers with percentages in parentheses where appropriate. Quantitative variables were reported as mean with standard deviation or with minimum and maximum values, depending on the specific variable.

## 3. Results

**Figure 1:**
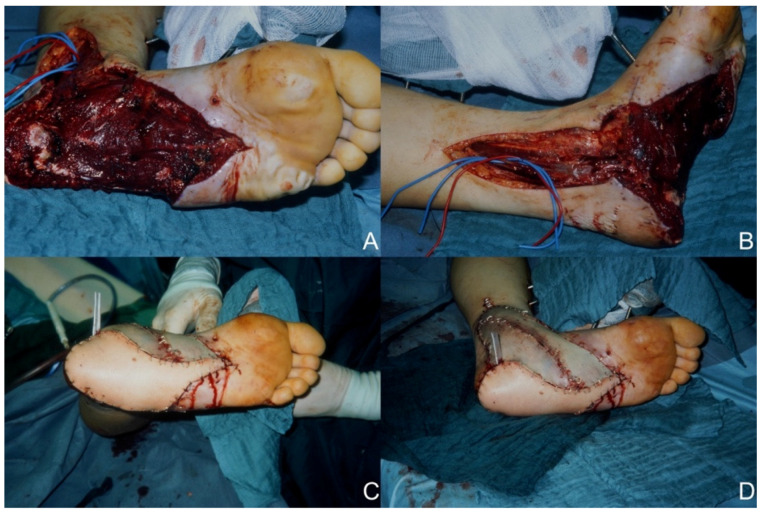
Reconstruction of an extensive foot defect using a latissimus dorsi flap. (**A**) Preoperative defect plantar view. (**B**) Preoperative defect medial view. (**C**) Postoperative plantar view. (**D**) Postoperative medial view.

A total of 25 patients could be included in the study (Table 1). Of these, 20 (80%) patients were men, and 5 (20%) patients were women. The mean age of the patient group was 40.3 ± 19.1 years. The most common comorbidity was diabetes mellitus with seven patients affected (28%), followed by peripheral arterial disease (*n* = 5/20%), hypertension (*n* = 3/12%), drug abuse (*n* = 2/8%), and smoking (*n* = 2/8%) as comorbidities. The average defect size was 127 cm^2^ (range: 20 cm^2^–400 cm^2^). For cases with trauma as the etiology, the average time between trauma and surgical care was 32.8 ± 43.6 days. The defect site involved the heel in 12 cases (48%), the plantar midfoot in one case (4%), and the plantar forefoot in two cases (8%). The dorsum of the foot was affected in nine (36%) and the ankle in eight (32%) defect situations. The sum of defect locations exceeded the sum of microsurgical procedures since in some defect situations multiple regions of the foot were involved. A defect situation after high-speed trauma and images of the postoperative situation after latissimus dorsi flap reconstruction is shown in Figure 1.

The etiology of the reconstructed defects in descending frequency was: trauma (*n* = 17/68%), ischemia (*n* = 4/16%) infection (*n* = 3/12%), and burns (*n* = 1/4%). The patient group underwent a total of 27 free flap reconstructions (one patient received three free flap procedures). The latissimus dorsi flap (*n* = 13/52%) was the most used reconstructive technique. The other free flap techniques used were: anterolateral thigh flap (*n* = 8/32%), parascapular flap (*n* = 3/12%), gracilis flap (*n* = 2/8%), and radial forearm flap (*n* = 1/4%). Most traumatic defect situations (9 of 13) were restored using latissimus dorsi flaps as was our only burn injury case. Two latissimus dorsi flaps and two anterolateral thigh flaps were used in the four defect situations after ischemia. For cases of infection as defect, one latissimus dorsi flap, one anterolateral thigh flap, and two gracilis flaps were used. A comparison of the etiology of the defects and the reconstructive procedures used for them can be seen in Table 2. The most frequently used recipient artery was the posterior tibial artery (twelve anastomoses). The anterior tibial artery was used in eleven procedures, the dorsalis pedis artery in three, and the peroneal artery in one.

**Figure 2:**
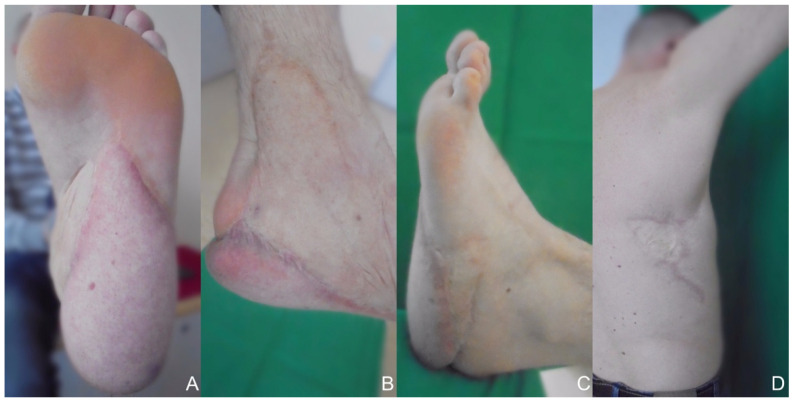
Healed donor and recipient site 20 years after reconstruction at a follow-up meeting. (**A**) Inferior view. (**B**) Medial view. (**C**) Lateral view. (**D**) Donor site.

Postoperative complications after reconstructive surgery were analyzed. Complications occurred in a total of 18 patients. Minor deviations from the normal postoperative course (e.g., increased need for analgesics) were also assessed. No postoperative complications occurred in seven patients. A total of 23 complications occurred, 15 (65%) of which required surgical intervention. Two of the three total flap losses involved the two gracilis flaps and the other total flap loss involved a patient who received a latissimus dorsi flap reconstruction for the sole of the foot. Based on total flap losses, the success rate is 92% in terms of the number of patients we treated and 89% in terms of the number of successful reconstructive surgeries. A summary of complications according to the Clavien–Dindo classification is shown in Table 3 [12]. In addition to the complications, two patients required three debulking surgeries each. The affected patients had both received a parascapular flap on the dorsum of the foot. The time of follow-up for the remaining patients ranged from two months to 20 years. Functional outcome was predominantly good; however, many of the patients required modified footwear after reconstruction. Figure 2 shows a healed donor and recipient region after latissimus dorsi flap reconstruction at a 20-year follow-up meeting.

## 4. Discussion

In the past, extensive foot defects were always indications for amputation. In addition to the obvious functional deficit caused by major limb amputation, limb loss also creates a significant psychological burden for affected patients [13]. Advances in microsurgical reconstruction with free flaps now provide expanded options for limb salvage. McCarthy et al. clarified in 1999 that the additional technique of free flap with revascularization is an alternative to amputation for ischemic foot wounds [14]. Langstein et al. also stated in 2002 that free flaps support limb preservation in malignancies of the foot [15]. Nevertheless, based on the long-term results, it is debatable whether reconstruction is always the best solution [16]. Krettek et al. stated 2016 that patients with severe trauma of the foot who require a free flap reconstruction have a significantly worse outcome than patients undergoing a below-knee amputation [17]. In a situation where it is unclear whether limb preservation should be attempted, such as in cases of complex defects of the foot, it seems appropriate to look at scales for reasonable decision-making. However, Bosse et al. concluded in their multicenter study that scoring systems for lower limb injuries (e.g., Limb salvage Index or NISSSA-score) do not provide a significant benefit for the initial decision as to whether limb salvage should be preferred [17,18]. Thus remains the surgeon’s clinical evaluations of the best therapeutic option, which must always consider the patient’s wishes. These, of course, often lean strongly toward limb preservation, as in the patients we have operated on.

In our study, reconstruction of larger foot defects using microsurgical reconstruction proved to be a promising alternative to limb amputation with a success rate of 89%. Both latissimus dorsi flaps and anterolateral thigh flaps showed good results in our study, with 12 of 13 and 8 of 8 successful surgeries, respectively, and proved to be reliable reconstruction methods for larger defect situations that may include the weight-bearing area of the foot. In our study, so as to provide an objective basis for decision-making, we recorded complications, including minor ones, using a complication classification system [12]. The most common complications in the cases we studied were wound healing complications, which required debridement and, if necessary, covering by a skin graft. In a comparative study, free flaps had the highest success rate but also the highest reoperation rate compared to local tissue rearrangements and skin substitutes [19]. However, the frequent complications and increased number of follow-up surgeries after free flap procedures also highlight the limitations and drawbacks that patients might have to face.

Follow-up meetings were scheduled for wound control and monitoring of the functional outcome. The follow-up was uneventful and stable wound conditions were observed. We graded the patient who underwent surgery at the University Medical Center Schleswig-Holstein, Campus Lübeckin 2000, with the 20-year follow-up meeting at Klinikum Nürnberg according to the AOFAS score for evaluating outcome after surgical procedures presented by Kitaoka et al. [20]. After 20 years, this patient scored 87 points out of 100, with the main drawbacks being related to pain, with mild temporary pain in the lateral sole area. The patient is also able to wear regular shoes and play football again. After successful defect coverage, all patients in our study underwent rehabilitation to regain their walking ability. Nevertheless, an asymmetry in the gait remains detectable in almost every case [21]. In total, postoperative follow-up was not undertaken for eight patients, partly due to the fact that these patients came from further away to have the operation performed in our department. The need to perform debulking procedures at a later stage is not uncommon and has already been reported in other publications [10,22,23].

The indication for the reconstruction of a complex plantar defect still presents the surgeon with an extraordinary challenge. The choice of flap should be based on the size of the defect, depth of the defect, exact location of the defect, donor tissue options, and the surgeon’s experience [3,24]. In the systematic review by Crowe et al., the latissimus dorsi flap was the most used free flap, as it was in the cases we operated on [3]. Furthermore, in this systematic review, the latissimus dorsi flap is the surgical technique used to repair the largest defect situations. This is consistent with our experience in our study, in which we successfully used the latissimus dorsi flap for our largest defect situation (400 cm^2^). A concomitant reinnervation of the flaps is not mandatory, since the protective sensitivity (which is favorable for flap survival) is usually achieved after 12 months even without a reinnervation procedure [25,26]. Potparic et al. examined complications and functional outcome after reinnervated and non-innervated reconstruction of the plantar foot using free flaps [11]. No significant difference could be found during the case series. When considering the correct timing for defect coverage, immediate debridement followed by reconstruction is recommended (especially in traumatic wounds) [27]. According to the classification of free flap reconstructions by Ninkovic et al., three types of free flap closure can be distinguished based on the timing of reconstruction after injury: primary (12 to 24 h), delayed primary (2 to 7 days), and secondary (after 7 days) [28]. In our study, most patients with defects after trauma had suffered extremely severe injuries, and osteosynthesis and further operations were often required initially to create a surgical site suitable for a free flap transfer. Due to this fact, primary flap coverage was not possible in any of our cases, and 12 cases are secondary free flap closures.

In our clinic, the frequency of free flaps for reconstruction of foot defects has decreased due to the possibility of reconstructing complex defects, even in weight-bearing areas (such as the heel) using innovative treatment options from the field of dermal skin substitutes. A meta-analysis showed a reduced amputation rate in diabetic foot ulcers when treated with skin substitutes [29]. With NovoSorb^®^ Biodegradable Temporising Matrix (BTM) (Polymedics Innovations GmbH, Denkendorf, Germany), even deep wounds can be treated [30]. However, skin substitutes, such as BTM, also have their limitations, notably in cases of poor vascularization or possible infectious events in the defect area, for example [30]. Following these findings, there may be a future balancing act between novel skin substitutes and autologous reconstruction procedures for large defects of the foot [19]. Influencing factors, such as comorbidities, status of bones, and the threat of losing another limb in the near future, need to be considered before choosing the best treatment option for every individual patient [31]. In principle, however, flap reconstructions are suitable for threatened ischemic lower extremities (in combination with revascularization procedures), traumatic defects (including burns), tumors, and infections.

In our study, we presented a case series in the field of microsurgical foot reconstruction paying particular attention to expected complications and setbacks; and with our follow-up of up to 20 years, we also addressed achievable functional outcomes. Our study thereby integrates into the existing literature on this topic and, based on our described cases, provides guidance in decision-making regarding indications for foot reconstruction. Limitations of our study are the non-randomized protocol of the study design. Furthermore, the number of patients is small.

## 5. Conclusions

Although there are many reconstructive options for foot defects, in the field of reconstructive surgery, this remains one of the major challenges. In our study, reconstruction with free flaps proved to be a robust technique for the reconstruction of even extensive defects. Reconstruction is also possible in patients with concomitant diseases, such as diabetes and peripheral arterial disease, and generally in any type of defect. It must be added that complications and revision after these procedures are not uncommon and would have to be accepted for limb salvage. In the follow-up of our patients, the functional results after such reconstructions were predominantly good. More studies comparing the long-term results and the impact on the daily life of the patients should be undertaken to enable the formulation of evidence-based algorithms for flap selection; and of clinically proven decision-making criteria for identifying under which circumstances reconstruction using advanced surgical procedures is indicated.

## Figures and Tables

**Table 1:** Demographics, comorbidities, defect site, reconstructive procedure, and follow-up.

Age	Sex	Comorbidities	Time between Trauma and Surgery (Days)	Size of Defect (cm × cm)	Location of Defect	Type of Reconstructive Procedure	Recipient Artery	Duration of Follow Up
40	m	DM, PAD, HTN, Smoke		7 × 5	Heel	LD flap	PTA	
58	m				Plantar forefoot	RF flap	ATA	4 months
20	m		34		Heel	LD flap	PTA	
26	m		33	30 × 11	Dorsum of foot	LD flap	ATA	3 months
60	w		24	15 × 8	Ankle region	LD flap	PTA	9 months
44	m	Drug abuse	4	5 × 4	Ankle region	ALT flap	ATA	2 years
49	m	DM, PAD			Heel + Ankle region	LD flap	ATA	3 years
16	m		13	12 × 8	Heel	ALT flap	ATA	15 months
20	m		28	24 × 12	Heel	LD flap	PTA	16 months
31	m	Drug abuse		10 × 8	Dorsum of foot	ALT flap	ATA	
67	m	DM, PAD	5	15 × 8	Dorsum of foot + Ankle region	LD flap	PTA	
15	w		14	10 × 6	Dorsum of foot + Ankle region	PS flap	ATA	9 years
46	m	Smoke	156		Dorsum of foot	ALT flap	DPA	8 years
43	m			6 × 4	Ankle region	FG flap	ATA	
						FG flap	PA	
						LD flap	ATA	
17	w		8	16 × 8	Dorsum of foot	PS flap	ATA	3 years
72	m	DM, PAD, HTN		7 ×7	Heel	LD flap	PTA	5 months
10	m			16 × 7	Heel	LD flap	PTA	4 months
57	m	HTN	115	18 × 7	Heel	PS flap	PTA	2 months
39	w	DM	6	5 × 5	Heel	ALT flap	PTA	10 months
27	m		11	12 × 12	Dorsum of foot	LD flap	DPA	
71	m	DM		12 × 5	Heel	ALT flap	PTA	6 months
55	m		12	15 × 12	Dorsum of foot + Heel	LD flap	ATA	16 months
55	w		29	17 × 10	Dorsum of foot + Plantar forefoot	ALT flap	DPA	4 months
51	m	DM, PAD		12 × 9	Ankle region	ALT flap	PTA	
18	m			20 × 20	Heel, Midfoot + Ankle region	LD flap	PTA	20 years

DM: diabetes mellitus, PAD: peripheral arterial disease, HTN: hypertension, LD: latissimus dorsi, RF: radial forearm, ALT: anterolateral thigh, PS: parascapular, FG: free gracilis, ATA: anterior tibial artery, PTA: posterior tibial artery, DPA: dorsalis pedis artery, PA: peroneal artery.

**Table 2:** Type of reconstructive procedure and etiology of defect.

Reconstructive Procedure	Trauma	Ischemia	Infection	Burn Injury
Latissimus dorsi flap (13)	9	2	1	1
Anterolateral thigh flap (8)	5	2	1	0
Parascapular flap (3)	3	0	0	0
Gracilis flap (2)	0	0	2	0
Radial forearm flap (1)	1	0	0	0

**Table 3:** Postoperative complications.

Complications		Grade 1		Grade 2		Grade 3	
		N	%	N	%	N	%
Reconstructive site	Thrombosis					2	100
	Infection			1	50	1	50
	Wound healing complications			1	11	8	89
	Partial necrosis					1	100
	Full necrosis					3	100
Donor site	Seroma	2	100				
Other complications	Extreme pain	1	100				
	Fever	1	100				
	Anemia			2	100		
Total		4		4		15	

## Data Availability

Not applicable.
